# Metabolic indexes of obesity in patients with common mental disorders in stable stage

**DOI:** 10.1186/s12888-022-03752-2

**Published:** 2022-02-07

**Authors:** Xiaoling Li, Xiaojie Shi, Yukang Tan, Yang Yu, Chaohua Tang, Guohong Xu, Xinglian Zhang, Hairong Liao, Xiancong Mai, Wensheng Chen, Xin Luo, Caixia Xu, Guojun Xie, Jiaquan Liang

**Affiliations:** 1 Department of Psychiatry, The Third People’s Hospital of Foshan, Guangdong, People’s Republic of China; 2 Center On Translational Neuroscience, Minzu University of China, Beijing, People’s Republic of China

**Keywords:** Obesity, Body mass index, Major depressive disorder, Bipolar disorder, Schizophrenia

## Abstract

**Background:**

Obesity is a serious worldwide public health problem, especially for people with mental disorders.

**Aim:**

To explore the related factors of obesity by analyzing the metabolic indexes of patients with common mental disorders in stable stage.

**Methods:**

Five hundred seventy-six subjects with major depressive disorder (MDD), bipolar disorder (BD) or schizophrenia (SCZ) were included, who received fixed drug dose and routine drug treatment for 2 years or more. Their venous blood was collected, and the blood metabolic indexes were analyzed.

**Results:**

BD and SCZ are more prone to obesity than MDD. Multiple linear regression analysis showed that the value of BMI increased with the increase of age(*B* = 0.084, *p* < 0.001), TG(*B* = 0.355, *p* = 0.024), LDL(*B* = 0.697, *p* < 0.001), LDH(*B* = 0.011, *p* = 0.002), SCr(*B* = 0.051, *p* < 0.001), UA(*B* = 0.014, *p* < 0.001), HbA1c(*B* = 0.702, *p* = 0.004) and hsCRP(*B* = 0.101, *p* < 0.001). And It decreased with the increase of HDL(*B* = -1.493, *p* < 0.001).

**Discussion:**

People with mental disorders should regularly check blood indicators and strengthen weight management to reduce the risk of obesity and promote their health.

## Introduction

Obesity is a serious worldwide public health problem. It was found that the number of obese people in China currently exceeds 85 million through continuous surveys in rural and urban areas across the country [1]. According to Chinese standards, about 50% of adults and 20% of children are overweight or obese [2].

Previous studies have shown that there was no significant difference in the incidence of obesity in the first-episode untreated major depressive disorder (MDD) [3], bipolar disorder (BD) [4] and schizophrenia (SCZ)) [5] compared with healthy controls. However, after using psychotropic drugs for a period of time, the weight of patients would increase in varying degrees, even to the extent of obesity [6].

The rationale for examining metabolic factor is underdeveloped and the connection between the psychiatric disorders and obesity and metabolic factors is poorly described. Some studies have shown that age, education, smoking, drinking and other living habits are related to obesity [7]. In terms of blood metabolic indexes, total cholesterol [8], low density lipoprotein [9], uric acid [10] and glycosylated hemoglobin [11] are also related to obesity, too. However, there are few studies on the analysis of multiple indicators, and the results are inconsistent.

Our study collected blood from patients with common mental disorders, no smoking and drinking habits. In order to find the related influencing factors of obesity and provide basis for clinical intervention.

## Methods

### Participants

Outpatients with MDD, BD and SCZ were included in this study, who met the diagnostic criteria of the Diagnostic and Statistical Manual of Mental Disorders, Fourth Edition (DSM-IV) from May 2015 to October 2020 in the Third People's hospital of Foshan, Guangdong, China(Foshan is located in the northern part of the Pearl River, 20 km (12 miles) from Guangzhou, Guangdong Province). Their age was ≥ 18 years old, and they were required to maintain a fixed drug dose pattern for 2 years or more (in the stable period) before blood testing.

Exclusion criteria: ①Comorbidity and other mental disorders, including mental retardation or other cognitive impairment; ②Patients with severe and unstable physical diseases, including severe liver and kidney function damage, cardiac insufficiency, etc.; ③Smoking habits(≥ 1 cigarette per day) or drinking habits(≥ 1 unit alcohol per week); 1 unit alcohol = 480 ~ 600 ml of beer = 350 ml of low alcohol liquor or red wine, yellow wine = 50 ml of high spirits (that is 40 degrees or more); ④Those who cann’t cooperate with venous blood drawing, such as phobia, etc.

### Assessments

For the subjects who met the above conditions and were willing to participate in the study, after signed the informed consent, collected the names, gender and age of the subjects through interviews. The height and weight of the subjects were measured with Automatic Measuring Stadiometer BSM370 (smitechasia.com) and the BMI (kg / m^2^) of each subject was calculated.

Whether the subject was overweight or obese was defined, according to the diagnostic criteria of overweight and obesity proposed by the China Obesity working group (24 kg/m^2^ ≤ BMI < 28 kg/m^2^ as overweight, BMI ≥ 28 kg/m^2^ as obesity and BMI < 24 kg/m^2^ as non obesity) [12].

Before drawing the venous blood of the subjects, they were required to be fasting for more than 8 h, and the nurses were required to complete the blood drawing of them from 7:30 to 10:00 in the morning. The night before blood drawing, the subjects need to maintain a normal diet, follow the previous work and rest, do not exercise violently, do not drink alcohol or coffee after dinner. Total cholesterol (TC), triglyceride (TG), high density lipoprotein (HDL), low density lipoprotein (LDL), aspartate aminotransferase (AST), lactate dehydrogenase (LDH), creative kinase (CK), creative kinase isoenzyme (CK-MB), urea, serum creatinine (SCr), uric acid (UA), alanine aminotransferase (ALT), fast blood glucose (FBG), glycosylated hemoglobin (HbA1c), high sensitivity C-reactive protein (HsCRP) and homocysteine (Hcy) were recorded in the clinical data sheet of the subjects.

### Data analyses

Statistical Product and Service Solutions 21 software (SPSS 21, https://www.ibm.com/analytics/spss-statistics-software) was used to analyze the data. Chi square test was used to compare the differences of general demographic and blood metabolic indexes among MDD, BD and SCZ. The relationship between BMI and variable indexes was analyzed by Pearson correlation. Multiple linear regression was used to analyze the effects of blood index components on BMI.

## Result

### Comparison of demographic characteristics and metabolic indexes

A total of 576 subjects were included in this study, including MDD (*n* = 242), BD (*n* = 124) and SCZ (*n* = 210), while 24 subjects were excluded because of refusing to participate in the study, eating breakfast before blood drawing, or refusing to take medicine. There were no significant differences in age, sex, height, BMI, TC, TG, HDL, LDL, AST, CK, CK-MB, SCr, FBG, HbA1c and HsCRP among MDD, BD and SCZ (*p* > 0.05); And there were significant differences in body weight, BMI, LDH, urea, UA, ALT(U/L) and Hcy (*p* < 0.05). The incidence of obesity in MDD, BD and SCZ was 9.92%, 17.74% and 19.91% respectively. Overweight was 24.79%, 34.68% and 34.12% (Table 1).

**Table 1:** Comparison of demographic characteristics and metabolic indexes

	MDD(*n* = 242)	BD(*n* = 124)	SCZ(*n* = 210)	*F*	*p*
Age(year)	29.35 ± 13.41	28.49 ± 11.26	31.94 ± 11.62	0.584	0.774
Gender (male / female)	86/156	44/80	95/116	2.566	0.091
Height(cm)	163.19 ± 8.31	163.18 ± 8.28	164.02 ± 8.67	0.705	0.520
Weight^ac^	60.24 ± 12.26	64.75 ± 13.16	66.76 ± 12.72	15.680	< 0.001
BMI(kg/m^2^)^ac^	22.54 ± 3.79	24.27 ± 4.35	24.76 ± 3.97	19.026	< 0.001
Obesity(%)	9.92%	17.74%	19.91%	-	-
Over weight(%)	24.79%	34.68%	34.12%	-	-
Non obesity(%)	65.29%	47.58%	45.97%	-	-
TC(mmol/L)	4.985 ± 1.099	4.956 ± 0.952	4.995 ± 1.003	0.058	0.946
TG(mmol/L)	1.330 ± 0.813	1.442 ± 0.931	1.536 ± 0.970	2.703	0.053
HDL(mmol/L)	1.519 ± 0.395	1.482 ± 0.376	1.435 ± 0.441	2.564	0.092
LDL(mmol/L)	2.681 ± 0.812	2.711 ± 0.779	2.780 ± 0.721	0.954	0.388
AST(U/L)	20.562 ± 9.353	20.968 ± 12.710	27.457 ± 14.856	2.648	0.074
LDH(U/L)^ab^	167.814 ± 34.010	166.395 ± 40.264	181.305 ± 47.660	8.169	< 0.001*
CK(U/L)	113.537 ± 88.854	203.734 ± 69.657	137.486 ± 64.490	1.977	0.140
CK-MB(U/L)	12,417 ± 4.147	12.722 ± 7.697	13.089 ± 5.525	0.876	0.442
Urea(mmol/L)^c^	4.446 ± 1.252	4.400 ± 1.131	4.183 ± 1.073	2.985	0.047*
SCr(μmol/L)	63.479 ± 14.643	63.927 ± 12.633	65.600 ± 15.580	1.296	0.286
UA(μmol/L)^ac^	342.793 ± 100.554	376.427 ± 98.456	393.762 ± 114.681	13.912	< 0.001*
ALT(U/L)^ab^	25.112 ± 13.412	25.484 ± 26.888	35.205 ± 64.284	3.567	0.031*
FBG(mmol/L)	5.593 ± 2.309	5.373 ± 1.109	5.648 ± 1.410	0.968	0.384
HbA1c(%)	5.315 ± 0.638	5.233 ± 0.485	5.360 ± 0.635	1.640	0.185
HsCRP(mg/L)	2.550 ± 4.350	2.857 ± 5.369	3.532 ± 6.041	1.974	0.134
Hcy(μmol/L)^c^	11.895 ± 7.434	12.582 ± 8.931	14.280 ± 12.072	3.566	0.031*

*TC* total cholesterol, *TG* triglyceride, *HDL* high density lipoprotein, *LDL* low density lipoprotein, *AST* aspartate aminotransferase, *LDH* lactate dehydrogenase, *CK* creatine kinase, *CK-MB* creatine kinase isoenzyme, urea, *SCr* serum creatinine, *UA* uric acid, *ALT* alanine aminotransferase, *FBG* fasting blood glucose, *HbA1c* glycosylated hemoglobin, *HsCRP* hypersensitive *C*-reactive protein, *Hcy* homocysteine, a: MDD vs BD, b: BD vs SCZ, c: MDD vs SCZ, *p* < 0.05; * indicates the comparison between groups (*p* < 0.05); Values are expressed as mean ± standard deviation

### Correlation between BMI and various indexes

The results of Person correlation analysis showed that BMI was positively correlated with age, TC, TG, LDL, AST, LDH, SCr, UA, alt, FBG, HbA1c and hsCRP, and negatively correlated with HDL (Table 2).

**Table 2:** Correlation between BMI and various indexes

BMI								
Age*	*r*	0.331	LDH*	*r*	0.234	ALT*	*r*	0.221
	*p*	< 0.001		*p*	< 0.001		*p*	< 0.001
TC*	*r*	0.225	CK	*r*	0.020	FBG*	*r*	0.155
	*p*	< 0.001		*p*	0.638		*p*	< 0.001
TG*	*r*	0.335	CK-MB	*r*	0.030	HbA1c*	*r*	0.306
	*p*	< 0.001		*p*	0.469		*p*	< 0.001
HDL*	*r*	-0.250	Urea	*r*	-0.011	HsCRP*	*r*	0.262
	*p*	< 0.001		*p*	0.789		*p*	< 0.001
LDL*	*r*	0.340	SCr*	*r*	0.093	Hcy	*r*	0.038
	*p*	< 0.001		*p*	0.026		*p*	0.362
AST*	*r*	0.099	UA*	*r*	0.412			
	*p*	0.018		*p*	< 0.001			

*TC* total cholesterol, *TG* triglyceride, *HDL* high density lipoprotein, *LDL* low density lipoprotein, *AST* aspartate aminotransferase, *LDH* lactate dehydrogenase, *CK* creatine kinase, *CK-MB* creatine kinase isozyme, urea, *SCr* serum creatinine, *UA* uric acid, *ALT* alanine aminotransferase, *FBG* fasting blood glucose, *HbA1c* glycosylated hemoglobin, *HsCRP* hypersensitive *C* reactive protein, *Hcy* homocysteine, * indicates *p* < 0.05

### Multiple linear regression analysis of influencing factors of BMI

Taking BMI as dependent variable (*Y*) and age, TC, TG, HDL, LDL, AST, LDH, SCr, UA, Alt, FBG, HbA1c and HsCRP as independent variables (*X*), gender as covariate, a stepwise multiple linear regression model (*F* = 44.792, *p* < 0.001) was established for analysis. Finally, the elements entering the model were age, TG, HDL, LDL, LDH, SCr, UA, HbA1c and HsCRP (Table 3).

**Table 3:** Multiple linear regression analysis of influencing factors of BMI

Model	*B*	Standard error	Standard coefficient	*t*	*p*
(constant)	13.399	1.452	-	9.228	< 0.001
Age	0.084	0.012	0.253	7.146	< 0.001
TG	0.355	0.157	0.083	2.258	0.024
HDL	-1.493	0.341	-0.149	-4.379	< 0.001
LDL	0.697	0.191	0.131	3.656	< 0.001
LDH	0.011	0.003	0.107	3.163	0.002
SCr	0.051	0.010	-0.183	-5.008	< 0.001
UA	0.014	0.001	0.359	9.282	< 0.001
HbA1c	0.702	0.239	0.104	2.931	0.004
HsCRP	0.101	0.026	0.129	3.861	< 0.001

*TG* triglyceride, *HDL* high density lipoprotein, *LDL* low density lipoprotein, *LDH* lactate dehydrogenase, *SCr* serum creatinine, *UA* uric acid, *HbA1c* glycosylated hemoglobin, *HsCRP* hypersensitive *C* reactive protein

## Discussion

Our study included MDD, BD and SCZ patients who had treated with fixed doses of drugs for 2 years or more, and excluded smoking and drinking habits. The metabolism indexes were included in the analysis to explore the influencing factors of obesity, so as to provide reference for further clinical intervention.

The subjects of our study came from southern cities in China. According to a large-scale national epidemiological survey, the results show that the prevalence of obesity in urban areas in southern China was 2.8–7.2% from 2010 to 2018 [1]. Our results showed that the incidence of obesity in MDD, BD or SCZ is higher than the above data. This indicated that patients who regularly use psychotropic drugs for a long time have a higher incidence of obesity. What’s more, the possibility of obesity and overweight in BD and SCZ was higher than MDD, which is similar to the results of previous studie [13]. Clinically, the drug combination in BD and SCZ is more often, and the weight gain caused by mood stabilizer and antipsychotics is also difficult to avoid. Therefore, doctors need to pay more attention to the weight management in routine treatment [14].

The results of this study suggested that age, TG, LDL, LDH, SCr, UA, HbA1c and HsCRP were positively correlated with BMI, HDL was negatively correlated with BMI, which finally entered the multiple linear regression model. We found that age was a risk factor for obesity. With the increase of age, the risk of obesity will also increase. However, a previous study suggested that male's BMI decreases with age, while female's BMI increases with age [15]. This partially overlapped with our findings, which might be related to regional differences when we included subjects.

We also found that BMI was positively correlated with TG and LDL. Their effects on obesity have been unanimously recognized. It is generally believed that their increase is strongly related to the occurrence of obesity [16]. Therefore, in patients with stable mental disorders, regular monitoring of TG and LDL value is of great significance to prevent obesity.

LDH is an important enzyme in the process of glycolysis. And it is an important indicator of liver function together with ALT. Some studies had found that overweight and obesity were related to the increase of biochemical indexes of liver injury (LDH and ALT), which were involved in the occurrence of obesity [17].

Serum creatinine is the product of human muscle metabolism. It is closely related to the total amount of muscle in the body and is not easily affected by diet. In muscle, creatine slowly forms creatinine mainly through irreversible non enzymatic dehydration reaction, which is then released into the blood, filtered through glomerulus, and almost all of it is discharged with urine. Clinically, the detection of serum creatinine is an important index of renal function. The increase of serum creatinine means the damage of renal function. When studying longitudinal studies on diabetes, researchers found that the decrease of renal function was positively correlated with obesity [18]. In addition, uric acid is also metabolized through the kidney. Some people suggested that its increase was related to the increase of body weight and blood pressure [19]. Higher UA is significantly associated with increased risk of diabetes, especially among overweight people [20]. HbA1c reflects the average blood glucose level of the body for three months, and it is also an important indicator of diabetes diagnosis. Obesity often occurs in the early stages of diabetes, so HbA1c is easily understood by us as a risk factor for obesity. For patients with stable mental disorders, regular monitoring of SCr, UA and HbA1c can prevent the occurrence of complications.

HsCRP is an acute phase protein synthesized by hepatocytes during inflammatory stimulation such as tissue injury. As a member of inflammatory factors, it participates in the disease process of MDD, BD and SCZ [21]. Cho s et al. believed that it is associated with obesity at the same time [22]. Therefore, our study verifies the previous research and achieves good consistency.

All in all, it is not surprising that the above indicators are abnormal, but these indicators are related to the patient's liver function, kidney function, cardiac function and various lipid metabolism in the body, which is related to the in vivo action pathway of various psychotropic drugs. Therefore, in patients with long-term medication, testing these indicators will help to timely understand the physical health status, make corresponding intervention measures in advance, or adjust drug use to avoid obesity and other health problems.

Unfortunately, our study ruled out the patients with smoking and drinking habits, which might cause partial data deviation. Although many previous studies suggest that there is no difference in the incidence of obesity between the first-episode untreated patients with mental disorders and healthy controls, the causal relationship between obesity and various indicators still needs to be further explored. Therefore, we still need to be cautious in the promotion of follow-up conclusions.

In conclusion, patients with stable mental disorders should strengthen the monitoring of relevant blood indicators and weight management, so as to reduce the risk of obesity and promote their health.

## Data Availability

The datasets generated and/or analyzed during the current study are not publicly available due to confidentiality but are available from the corresponding author on reasonable request.
